# A State-Transition-Free Delayed-Feedback Task Elicits Heterogeneous Human Responses

**DOI:** 10.5334/joc.453

**Published:** 2025-07-14

**Authors:** Satoshi Hirata, Yutaro Sato, Hika Kuroshima, Yutaka Sakai

**Affiliations:** 1 Wildlife Research Center, Kyoto University, Kyoto, Japan; 2 University Administration Office, Niigata University, Niigata, Japan; 3 Graduate School of Letters, Kyoto University, Kyoto, Japan; 4 Brain Science Institute, Tamagawa University, Tokyo, Japan

**Keywords:** Reinforcement learning, Delayed reward, Credit assignment problem, Eligibility trace, Explicit learning

## Abstract

Humans and nonhuman animals learn to perform actions by associating actions with outcomes. In everyday life, outcomes sometimes occur only after a delay, and at an unexpected moment. The ability to connect actions and delayed outcomes has received less attention than performance in tasks where rewards follow the most recent action. Here, following a previous study (Sato et al. 2023), we designed a learning task to investigate humans’ ability to link actions and outcomes which occurred after intervening choices. We prepared a total of six visual stimuli for use in three types of trials: A vs B, where choosing A immediately led to reward and choosing B was never rewarded, C vs D, where neither choice was immediately rewarded but choice of C led to reward in a later E vs F trial, and E vs F, where neither stimulus was associated with reward but a reward was given based on choice of C in the past. Results showed that nine individuals learned to choose C, thereby receiving a delayed reward. Among them, one participant subsequently correctly described the task structure in words, while the remaining eight did so with misunderstandings. We also observed large individual differences in participants’ action selection (e.g., an irrational bias for D, a possible superstitious bias for either E or F) and explicit/implicit understanding of the link between action and delayed outcome expressed in words. Our results offer new insights into the ability to cognitively link actions and outcomes following a time lag.

## Introduction

Humans and nonhuman animals learn to perform actions by associating the action with its outcome (Skinner 1938). Studies of learning often use tasks in which the outcome (e.g., food reward, feedback for answers) immediately follows the action (e.g., pressing a lever, choice of correct visual stimulus). However, in daily life, the outcome of an action does not necessarily occur immediately after the action, but instead may occur at a later, unexpected moment (Jocham et al. 2016; Lehmann et al. 2019). For this reason, individuals need to properly associate the outcome and the past causal action, a credit assignment problem (Minsky 1961).

Some studies on humans’ responses to delayed rewards have used a two-step task (e.g., Daw et al. 2011; Akam et al. 2015), in which the participant’s choice in the first step does not lead to any immediate reward but changes the probabilistic structure of the second step. Most such studies have applied the reinforcement learning computational model incorporating an eligibility trace (Singh & Sutton 1996; Sutton & Barto 2018). The eligibility trace is a mathematical parameter in reinforcement learning algorithms that makes a bridge between a past action and current reward. Studies using this approach have generally supported the view that the eligibility trace explains the behavior of humans as well as nonhumans (Daw et al. 2011; Akam et al. 2015; Miranda et al. 2020). Another approach involves inserting a delayed reward delivery in a cognitive task. Smith et al. showed that humans have difficulty in learning when feedback for Trial N arrives after Trial N+1; feedback was delayed by one trial, in the “1-back” task (Smith et al. 2018). Prior to the test participants were told that their feedback would always lag one trial behind, leaving it unclear whether they could understand the link between an action and delayed feedback without prior instruction. Zentall et al. (2023) used the 1-back task to investigate learning of symbolic matching. About half of the participants did not learn, indicating that the 1-back task can be difficult. When asked to describe the rule of the task, no participants mentioned the 1-back rule, even among those who learned the symbolic matching task. The researchers’ focus in their study was on conscious learning processes, contrasting explicit learning (including hypothesis testing) and rule formation using executive functioning (e.g., working memory) or on implicit learning, including gradually forming an association between stimulus and response based on repeated experience (Church et al. 2021).

**Figure 1:**
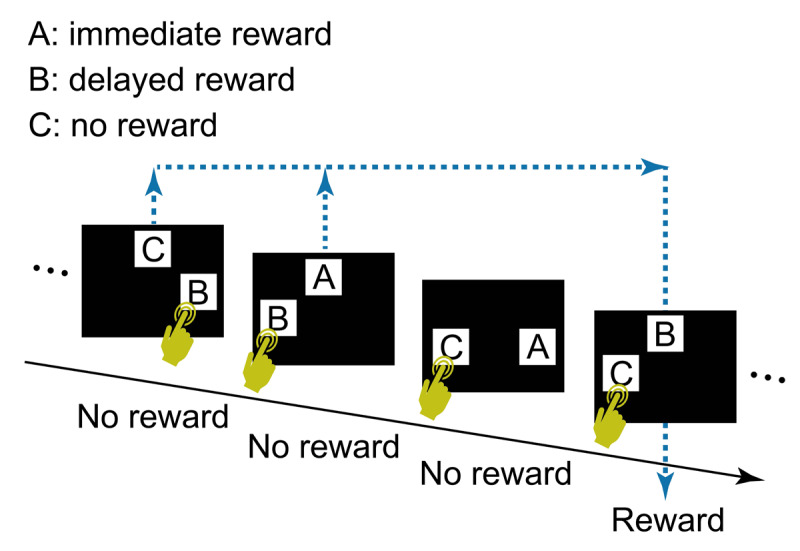
The task design used by Sato et al. (2023).

Under a computational theory of reinforcement learning (Sutton & Barto 2018), typical cognitive tasks in which outcome of an action follows the action immediately can be formulated as follows: the state transition (e.g., from an action phase to a feedback phase) and reward (e.g., a piece of food) depend solely on the most recent state (e.g., the action phase) and action (e.g., a lever press), i.e., a Markov property (Howard 1960). The ability to connect an action and its delayed outcome in a non-Markov property is less well studied than in tasks with Markov decision processes (Walsh & Anderson 2011). Sato et al. (2023) designed a touch-screen task to examine chimpanzees’ ability to associate their choice of visual stimuli with a delayed reward, using a simplified version of Tanaka et al.’s (2009) task (Figure 1). On each trial the chimpanzee chose one of two stimuli that were randomly selected from three stimulus types: A) associated with immediate delivery of a food reward, B) associated with reward delivery after a few intervening trials, and C) associated with no reward. If the chimpanzee touched B, no reward (food) arrived immediately, but a reward arrived when B appeared next in a B vs C trial regardless of the chimpanzee’s choice in that trial. Trials with different combinations of these three types of stimuli appeared randomly, i.e., there was no fixed pattern of trial sequence. In Sato’s et al. (2023) formulation, the reward and the upcoming state (the next trial) did not depend on the most recent state and action (i.e., current trial and stimulus choice, respectively), thus entailing a non-Markov property. In addition, the next trial type was independent of the participant’s choice in the current trial, making learning based on the step-by-step chain of state transition difficult. As this task favors learning processes free from computations based on state transitions, it can be called a delayed feedback task for state-transition-free learning (Sato et al. 2023). Of five chimpanzees tested, two learned to touch B and not C, suggesting some understanding of the link between choosing B and the delayed reward.

However, it was possible that chimpanzees solved the task simply because choosing B sometimes led to an apparent immediate reward. That is, if the chimpanzee touched B in Trial N of B vs C, no reward arrived but a reward arrived when B vs C appeared next time, in Trial N+i. Here, if the chimpanzee chose B again, the food reward arrived immediately, i.e., an apparent immediate reward for choosing B in Trial N+i. The task structure did not allow to fully dismiss the effect of apparent immediate reward, limiting the evidence for state-transition-free learning (Sato et al. 2023).

Another confounding factor in Sato et al.’s (2023) task was the omission of rewards for redundant choices of stimulus B. This was done to limit the number of rewards to either one or zero per trial, thereby reducing the cognitive load when discriminating reward quantities. However, this reward-exclusion criterion when stimulus B was chosen on multiple trials before B vs C trials were presented may have led to a degradation of the relative value of B due to its reduced contingency with reward. For example, Figure 1 illustrates a case where B was selected twice in the first two trials, but only one food reward was given, in the fourth trial, when B vs C appeared. Consequently, over the course of the session, the number of rewards for choosing stimulus B was smaller than the number of times B was selected, while every choice of stimulus A was rewarded. This reduced reward value might explain why three chimpanzees failed to learn to choose B. A potential solution would be to adjust the trial sequence so that each choice of stimulus is paired with a corresponding delayed reward.

Here, we modified the task used by Sato et al. (2023) to address the aforementioned issues. Firstly, we prevented an apparent immediate reward, by introducing a new pair of stimuli. Specifically, we prepared a total of six visual stimuli for use in three types of trials: A vs B, where choosing A is followed by immediate reward and B is never rewarded; C vs D, where neither choice is immediately rewarded but choosing C leads to a reward in a later E vs F trial, where neither E nor F is causally associated with reward, but a reward is given for choosing C in a previous trial. Therefore, choice of C is associated with a delayed reward but there is never an apparent immediate reward in C vs D trials. To maximize rewards, the participant must learn to choose A against B, and C against D, whereas choice of E or F does not matter. We thus tested the ability to understand the link between choosing C and the delayed reward that arrives unpredictably in E vs F trials. Secondly, to prevent the degradation of the relative value of the stimulus associated with the delayed reward, we designed the task so that each C vs D trial corresponded directly with a later E vs. F trial. The three types of trials were presented in a quasi-random sequence, with the number of each trial type kept consistent in a session.

The task we developed is potentially applicable to nonhuman animals, our ultimate goal being comparative studies to investigate the evolution of cognition related to the understanding of delayed outcome. Here, we tested human participants, who were given no instruction about the structure of the task, similar to studies with nonhuman animals. We predicted that humans would learn to link an action and its outcome even when there is a lag between the two and when the state transition (i.e., the sequence of trials) is independent of their action, assuming that they could connect the memory trace of past actions to the current outcome as exemplified mathematically by reinforcement learning with eligibility trace (Singh & Sutton 1996; Daw et al. 2011; Sutton & Barto 2018). We also predicted that they could do so even in the absence of an explicit understanding of the task structure, as shown by Zentall et al. (2023) in the 1-back task. We examined this possibility by obtaining post-task written reports from participants about their understanding of the task, and comparing their reports with actual task performance.

## Method

### Main experiment

#### Participants

Forty undergraduate students, graduate students, or postdoctoral researchers of Kyoto University participated (age range 18–36 years; 16 males and 24 females). They each received a library card worth 500 Japanese Yen for participating, regardless of their performance. Prior to the task, instructions were given on a printed document that was also read out by the experimenter (*Supplementary information*), followed by explanations about consent to publish and the reward (the library card). The participant then signed an informed consent form. No instruction about the structure of the task was given.

#### Material

The test was run on a standard laptop computer using PsychoPy (version 2023.2.3 based on Python3.8). Stimuli were presented on a 15.6-inch color touch-screen (ASUS MB16AMTJ) positioned approximately 40 cm away from the participant. Participants were tested individually in a room, in a single session.

#### Stimuli and Procedure

The participant was given a series of two-choice decision-making trials. We used black-and-white arbitrary shapes as visual stimuli (*Figure S1* in *Supplementary information*), which were presented on the touch-screen. The screen was 1920 × 1080 pixels in size and the visual stimuli were 324 × 324 pixels (30% of the height of the screen). A set of six stimuli, each with a different role, was used, referred to as stimulus A, B, C, D, E, and F. To reduce any effect of visual properties of stimuli on participants’ choice (e.g., preference for a specific visual stimulus), we prepared 12 visual images in total and assigned the role of stimuli A–F to six of the 12 images. We divided the 40 participants into four groups of 10, to which we assigned different combination of the visual images and the roles of stimuli (i.e., four patterns of stimulus allocation: see *Table S1* in *Supplementary information*).

The six stimuli were grouped into three pairs, A vs B, C vs D, and E vs F, and the participant selected one of the two by touching it on the screen. In an A vs B trial, touching A was immediately followed by a reward, and touching B was not followed by a reward. The reward was the score “+1 point” appearing on the screen; the score “+0 point” was shown on the screen when there was no reward. In a C vs D trial, no immediate reward was given regardless of which stimulus was touched (i.e., “+0 point” was shown after the trial), but touching C led to a delayed reward at a later E vs F trial. In a E vs F trial, “+1 point” or “+0 point” was shown after touching either stimulus, depending on the participant’s choice of C or D in an earlier trial.

**Figure 2:**
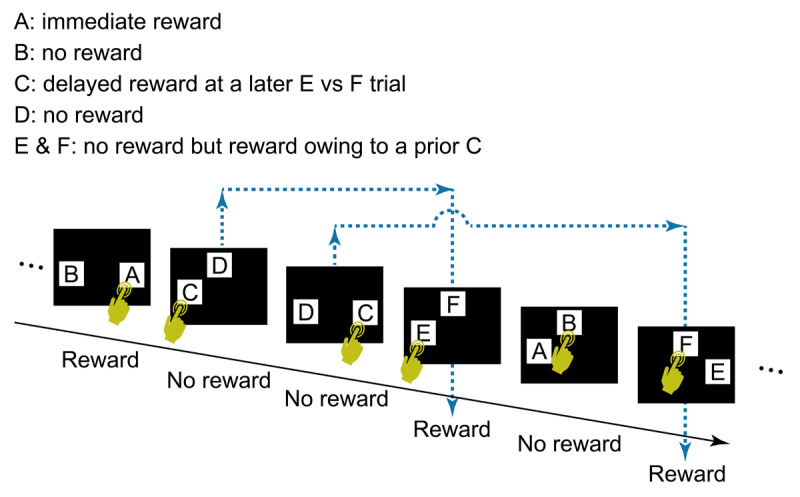
**The general task flow and structure**. In each trial, the participant touched a start button, a green rectangle that appeared at the bottom of the screen (not shown in Figure 2). The start button then disappeared and two visual stimuli (A and B, C and D, or E and F) appeared at two of three possible locations (i.e., upper center, lower left, or lower right), determined randomly. After the participant selected one stimulus by touching it, both stimuli disappeared, followed by display of the score (“+1 point” or “+0 point”) at the center of the screen for 2 s, after which the next trial began. A click sound was played for each touch of the start button and choice of a stimulus. The participant’s cumulative points total was shown continuously at the top right of the screen. Note that two C vs D trials could occur before a E vs F trial (see also Figure 3). In this case, the outcome of the E vs F trial reflects the choice in the first C vs D trial, not the second, the outcome of which is given in the subsequent E vs F trial. No more than two C vs D trials were presented before a E vs F trial.

**Figure 3:**
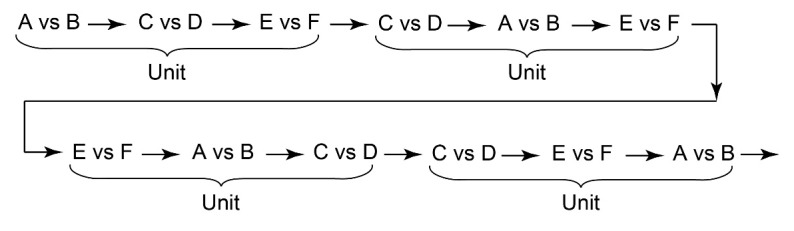
An example of the sequence of trials consisting of units containing three trials (A vs B/C vs D/E vs F).

The task flow and structure are illustrated in Figure 2. The order of three types of trials (i.e., A vs B, C vs D, E vs F) in a session was arranged so that each trial type appeared equally frequently. Specifically, the sequence of trials consisted of units of three trials, one of each type. The order of the three trials was randomized within a unit (Figure 3). This design ensured that the number of C choices, associated with delayed rewards, matched the number of rewards per session, except in cases where the final selection of C occasionally went unrewarded due to the quasi-random structure of the trials (specifically, if the last C vs D trial occurred after the final E vs. F trial). Each session consisted of 501 trials, containing 167 units of A vs B, C vs D, and E vs F trials. However, a session was terminated if the participant selected C in 8 or more trials out of 10 consecutive C vs D trials for two consecutive bins (a bin = 10 consecutive C vs D trials, counting continuously from the start of the session). This termination criterion was added so that the participant’s understanding of the task could be explored immediately after the bias for choosing C was formed.

After completing the session, participants were asked to write about their impression of the task. They were first shown drawings of the six visual stimuli that were presented on the screen, labeled as A–F. The written question to the participants was: “What was the task? Please describe your impression about the task freely. Please use the labels A–F to refer to the visual stimuli.”

#### Data analysis

We divided trials according to which stimuli were presented (A vs B, C vs D, and E vs F) and separated them into bins comprising 10 trials for each of the three trial types. Because the session contained 501 trials consisting of 167 trials for each of the three types, the last bin contained 7 trials, except for participants who reached criterion of eight or more choices of C in two consecutive bins and whose session was thus terminated.

To examine the distribution of participants’ final stimulus choice rates, we created scatter plots of (P_C_, P_A_) and (P_C_, P_E_), where P_A_, P_C_ and P_E_ represent the weighted averages of the respective choices of A, C, or E during the last 30 trials:


\[
{\rm P_X}=\sum_{\tau = 0}^{29}0.9^{\tau}\ a_{\rm x}(T-\tau)/{\sum_{\tau = 0}^{29}}0.9^{\tau}
\]

*a*_X_(*t*) = 1, 0 indicates whether the choice at trial *t* was X (X = A, C, or E) or not. Weighted averages were used for scatter plots to make the values continuous for visualization.

Given the relevance of individual differences in participants’ patterns of selection of stimuli for understanding the complex nature of learning processes in temporal credit assignment problems, we attempted analyses of the data categorized in several ways. First, we used chi square tests to explore choice biases in C vs D trials with timespans broader than 10-trial bins, namely the first vs second halves of the session, and within the whole session we used binominal tests. Second, we ran the same analyses for the E vs F trials to clarify what made the task challenging. Third, we examined the effect of immediate rewards by analyzing the reward obtained immediately after selecting stimulus E vs stimulus F, using Fisher’s exact tests. Note that these parameters were chosen after the data were collected because we had no a priori predictions regarding individual differences. For all statistical tests, alpha was set at 0.05 (two-tailed). Since the analyses focused on individual choice patterns, no adjustment was made for multiple comparisons. We used R software v.4.2.2 for all statistical analyses.

### Behavioral results of typical reinforcement learning models

For comparison, we conducted computational simulations using typical reinforcement learning models to assess whether a reinforcement learning algorithm could solve the present task. We adopted two well-known algorithms, actor-critic and Q-learning, along with three representative sets of model parameters (Sutton & Barto, 2018). Choice probabilities for each stimulus pair (A vs B, C vs D, and E vs F) were calculated using a softmax function based on the policy parameters, *q*_A_, *q*_B_, … *q*_F_,


\[
\begin{array}{*{20}{c}}
{P\left\{ {a\left( t \right) = {\mathrm{A}}} \right\} = {{\left( {1 + {e^{\beta {q_{\mathrm{B}}}\left( t \right) - \beta {q_{\mathrm{A}}}\left( t \right)}}} \right)}^{ - 1}},}\\
{P\left\{ {a\left( t \right) = {\mathrm{C}}} \right\} = {{\left( {1 + {e^{\beta {q_{\mathrm{D}}}\left( t \right) - \beta {q_{\mathrm{C}}}\left( t \right)}}} \right)}^{ - 1}},}\\
{P\left\{ {a\left( t \right) = {\mathrm{E}}} \right\} = {{\left( {1 + {e^{\beta {q_{\mathrm{F}}}\left( t \right) - \beta {q_{\mathrm{E}}}\left( t \right)}}} \right)}^{ - 1}},}
\end{array}
\]

where parameter *β* ≥ 0 controls the degree of random exploration (*β* = 0 means completely random). The policy parameters were updated according to the result of each step *t*,


\[
\begin{array}{*{20}{l}}
{{q_{\rm X}}(t + 1) = {q_{\rm X}}(t) + \alpha {\eta _{\rm X}}(t)\ \delta (t),}\\
{{\eta _{\rm X}}(t) = \lambda {\eta _{\rm X}}(t - 1) + \left\{ {\begin{array}{*{20}{c}}
{1\ (if\ a(t) = {\rm X})}\\
{0\ (otherwise)}
\end{array},} \right.}
\end{array}
\]

where the learning rate *α* controls the learning speed, the trace scale *λ* controls the length of the past memory trace. The reward prediction error *δ*(*t*) is differently updated in actor-critic and Q-learning,


\[
\delta \left( t \right) = \left\{ {\begin{array}{*{20}{c}}
{r\left( t \right) + \gamma {v_{s\left( {t + 1} \right)}} - {v_{s\left( t \right)}},}&{\left[ {{\mathrm{actor - critic}}} \right],}\\
{r\left( t \right) + \gamma \mathop {\max }\limits_{{\mathrm{X}}\ {\mathrm{in}}\ {\mathrm{next}}} {q_{\mathrm{X}}}\left( t \right) - {q_{a\left( t \right)}}\left( t \right),}&{\left[ {{\mathrm{Q - learning}}} \right],}
\end{array}} \right.
\]

where *r*(*t*) = 1, 0 represents whether it was rewarded or not at step *t*, and the discount factor *γ* controls the scale of future prediction. The variable *v*_s_ represents the value of each state (option pattern *s* = {A,B}, {C,D}, {E,F}) in choice-independent manner, updated at every step as


\[
{{v}_{\left\{ {\rm X},{\rm Y} \right\}}}\left(t+1 \right)={{v}_{\left\{ {\rm X},{\rm Y} \right\}}}\left(t \right)+\alpha \left({{\eta}_{{\rm X}}}\left(t \right)+{{\eta}_{{\rm Y}}}\left(t \right) \right)~\delta \left(t \right).
\]

We selected four representative sets of model parameters: slow learning (*α* = 0.001, *β* = 4, *λ* = 0.5), high exploration (*α* = 0.1, *β* = 0.01, *λ* = 0.5), short trace (*α* = 0.1, *β* = 4, *λ* = 0.01) and long trace (*α* = 0.1, *β* = 4, *λ* = 0.5). The discount factor was fixed (*γ* = 0.5). Each simulation began with initial values of \[
{{q}_{{\rm X}}}\left(0 \right)={{\eta}_{{\rm X}}}\left(0 \right)={{v}_{s}}\left(0 \right)=0~\] for all X and *s*, and continued for 16 trial bins (3 trials × 10 × 16 = 480 trials). Simulations were repeated 200 times for each combination of algorithm and parameter set.

### Follow-up experiment

A follow-up experiment was conducted to examine whether prior knowledge of the task structure would facilitate learning. Ten new participants (4 males, 6 females; aged 22–29) were recruited from the Kyoto University Graduate School. The experimental conditions were identical to those of the main experiment, except that participants received prior instruction about the task structure (details provided in *Supplementary information*). Ethical approval and informed consent procedures were as in the main experiment.

The instruction emphasized that the task involved three stimulus pairs presented in random order: in one pair, one stimulus would yield an immediate reward; in another, one stimulus would lead to a delayed reward while the other would not; and in the third, a reward would only be received if the stimulus associated with the delayed reward had been chosen in an earlier trial. However, no information was provided regarding which specific stimulus served each role.

## Results

### Main experiment

**Figure 4:**
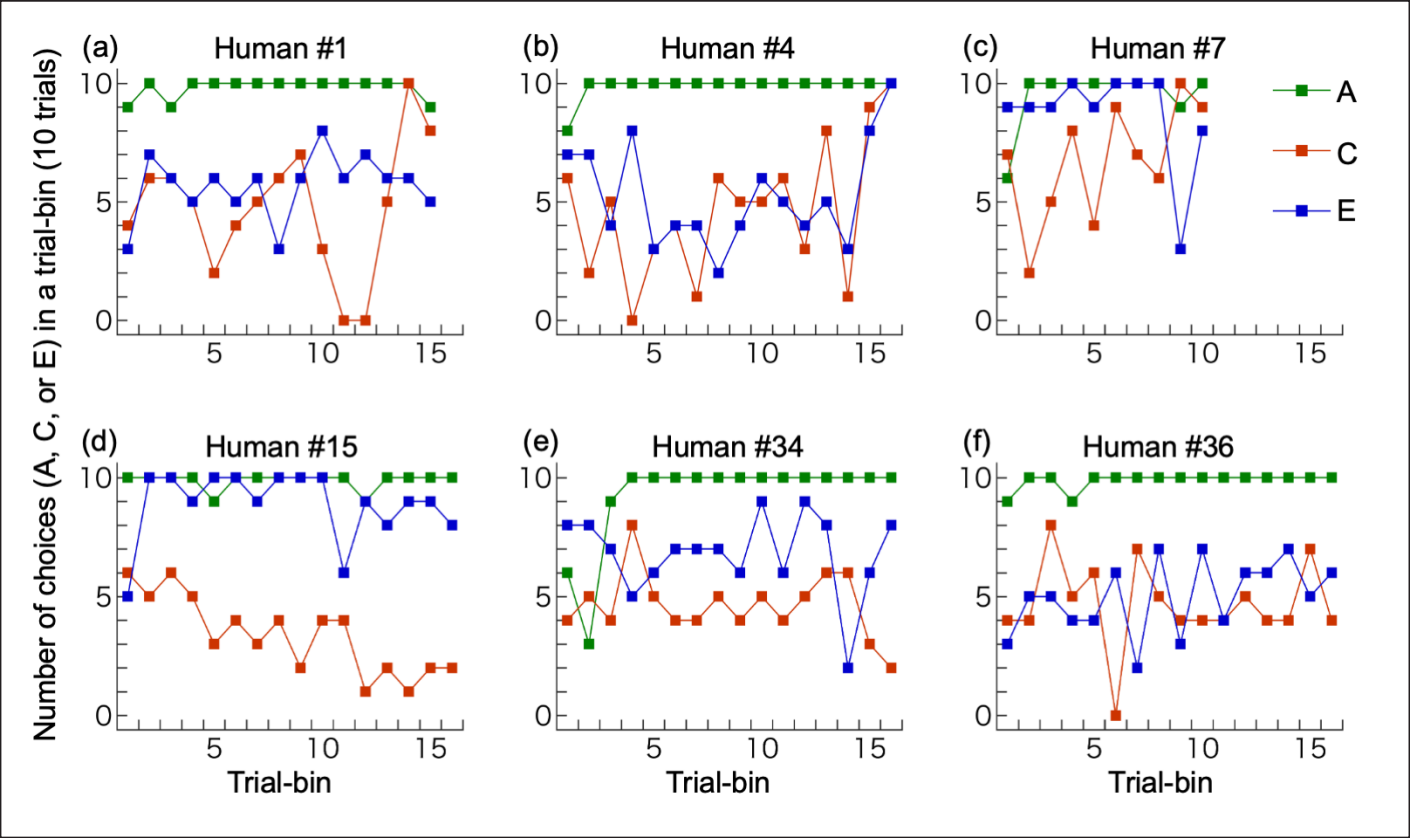
**Examples of individual performances. (a)** The participant with correct full understanding; **(b)** A participant with partial understanding; **(c)** A participant with overall bias for C, increase in C-choices in the latter half, and C-choices in ≥8/10 trials in two consecutive bins (which terminated the session). This participant did not write about the link between C vs D and E vs F trials; **(d)** A participant with overall bias for D, decrease in C (increase in D) in the latter half, and D-choices in ≥8/10 trials in more than two consecutive bins; **(e)** A participant with no bias for C or D but with a bias for E; **(f)** A participant with no bias for C or D and no bias for E or F. The last trial bin was not illustrated if it contained only 7 (not 10) trials: (d), (e) and (f).

**Figure 5:**
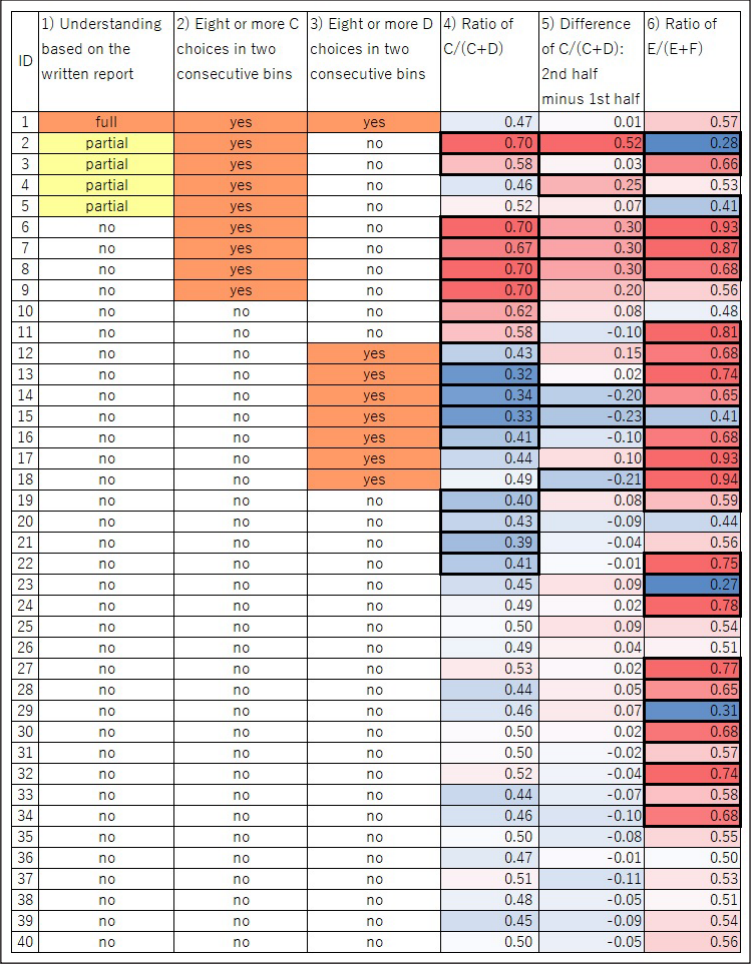
**Graphical table of the participants’ choices**. Columns 1)– 6) correspond to the categories described in the main text. For 4)–6), the intensity of the colors represents the degree of bias; red and blue in 4) and 6) mean that the value is more than 0.5 and less than 0.5, respectively; red and blue in 5) mean that the value is more than 0 and less than 0, respectively. Thick cell lines indicate a significant value in the cell (*p* < 0.05).

Contrary to our prediction, when data were pooled across participants, the overall proportion of choice C was 0.495, almost chance level (0.5). However, there were large individual differences among participants; Figure 4 shows performances of example participants. Figure 5 illustrates variations in participants’ performances according to the six criteria we developed after data collection was completed (*Supplementary information and Supplementary data* for more detailed results). We describe key findings below.

#### Written report

Only one of the 40 participants described the correct structure of the task in the written report after task completion (see *Supplementary data* for full comments). Four other participants’ descriptions were partially correct in that they mentioned the link between C vs D and E vs F trials, although some misunderstanding was evident, such as describing a link between D and F which did not actually exist. The remaining 35 participants made no mention of the link between C vs D and E vs F trials.

#### Choice of C in ≥8/10 trials in two consecutive bins

For nine participants the test was terminated before completing all 501 trials because they chose C in ≥8/10 trials in two consecutive bins containing 10 C vs D trials, the criterion we set to explore the participant’s subjective understanding of the task immediately after the bias for choosing C was formed. One and four participants (mentioned above) who wrote a correct or partially correct structure of the task, respectively were among these nine participants.

#### Choice of D in ≥8/10 trials in two consecutive bins

Eight participants chose D in ≥8/10 trials in two consecutive bins containing 10 C vs D trials. In contrast to the bias to C, this bias did not lead to termination of the session; participants continued to completion of all 501 trials.

#### Overall bias for C or D

A total of eight participants showed a significant bias (binominal test, *p* < 0.05) for C over D. Two of the eight showed partial understanding in their written report. In contrast, nine participants showed an overall bias for D over C.

#### Increase in the proportion of choices of C in the second half of the session

The frequency of choosing C increased in the second half of the session in 22 of the 40 participants; it decreased in 18. Chi-square tests revealed that four participants significantly increased (*p* < 0.05) their frequency of choosing C in the second half of the session, two of whom expressed partial understanding of the task in their written report. By contrast, three participants showed a significant change in the opposite direction (*p* < 0.05), namely an increased frequency of choosing D.

#### Significant bias in E vs F trials

There was no causal link between E or F and reward because a delayed reward due to a prior choice of C could be obtained for selecting either E or F. In addition, we counterbalanced the visual stimuli for E and F across participants, making it unlikely that any particular black-and-white shape used as a stimulus impacted participants’ selection. Nonetheless, 26 participants showed a significant bias for one or the other (*p* < 0.05).

#### Statistical significance of difference in the proportion of apparent immediate rewards in E vs F trials

Even if a participant chose randomly between C and D and between E and F, E might have been rewarded more frequently than F by chance. Alternatively, this might reflect the participant’s deliberate choice pattern. To determine the cause of the bias in E vs F trials, we analyzed differences in the proportion of apparent immediate rewards in E vs F trials. Of the 26 participants who showed a significant bias for E or F, four received significantly more delayed rewards immediately after selecting their preferred stimulus due to a sequence such as choosing E after C and F after D (*p* < 0.05). Two of them showed partial understanding of the task in their written report. In other words, 22 participants showed a significant bias for E or F but did not obtain significantly more immediate rewards by selecting their preferred stimulus (E or F).

### Performance of reinforcement learning models

**Figure 6:**
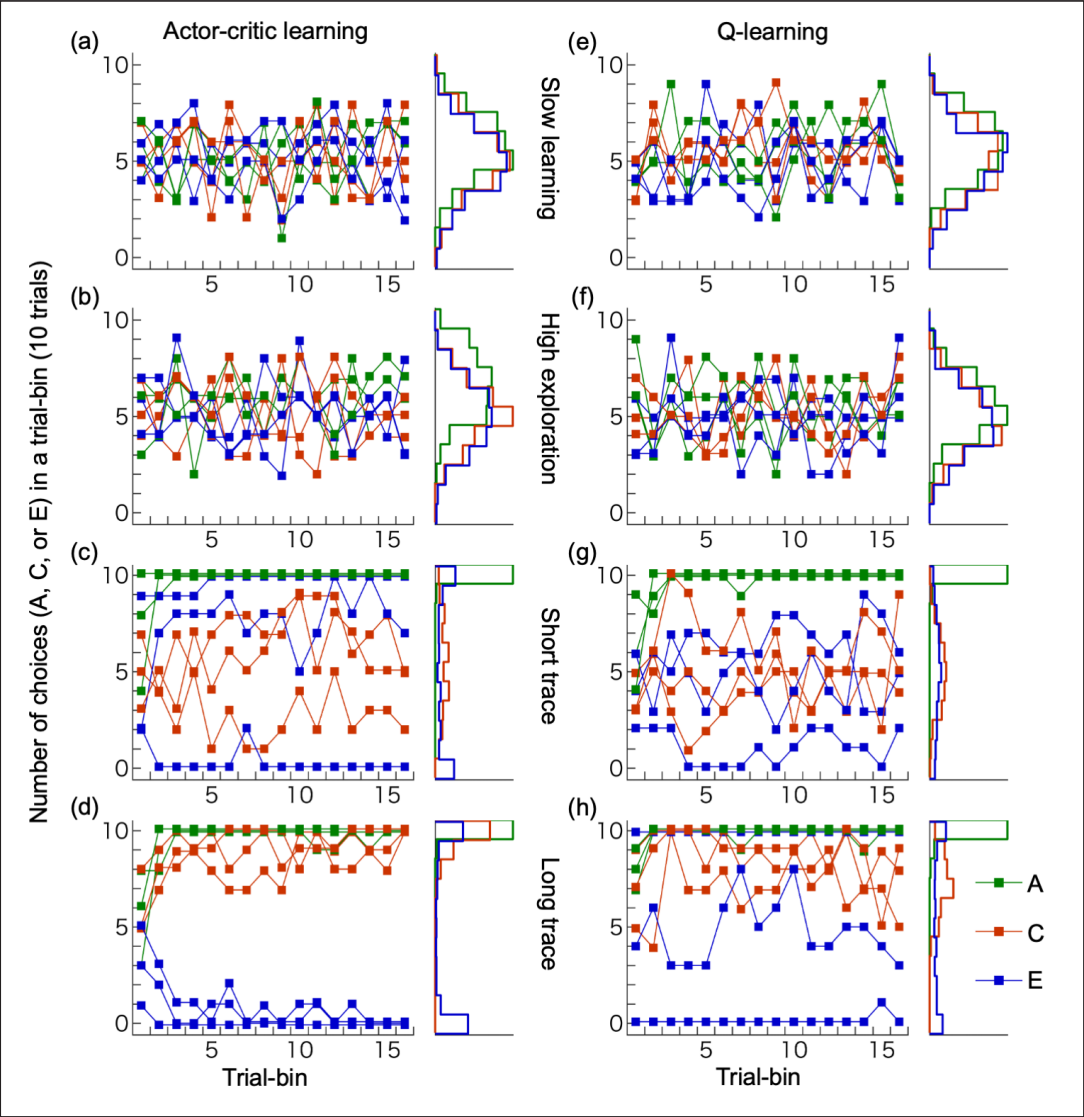
**Performances of reinforcement learning models**. **(a-d)** Actor-critic learning, **(e-h)** Q-learning. We implemented four types of learning parameters for simulations: slow learning **(a, e)**, high exploration **(b, f)**, short trace **(c, g)** and long trace **(d, h)**. For each parameter set, three example simulations are shown, along with histograms depicting the choice frequencies of stimuli A, C, and E in the final trial bin across 200 simulation runs.

**Figure 7:**
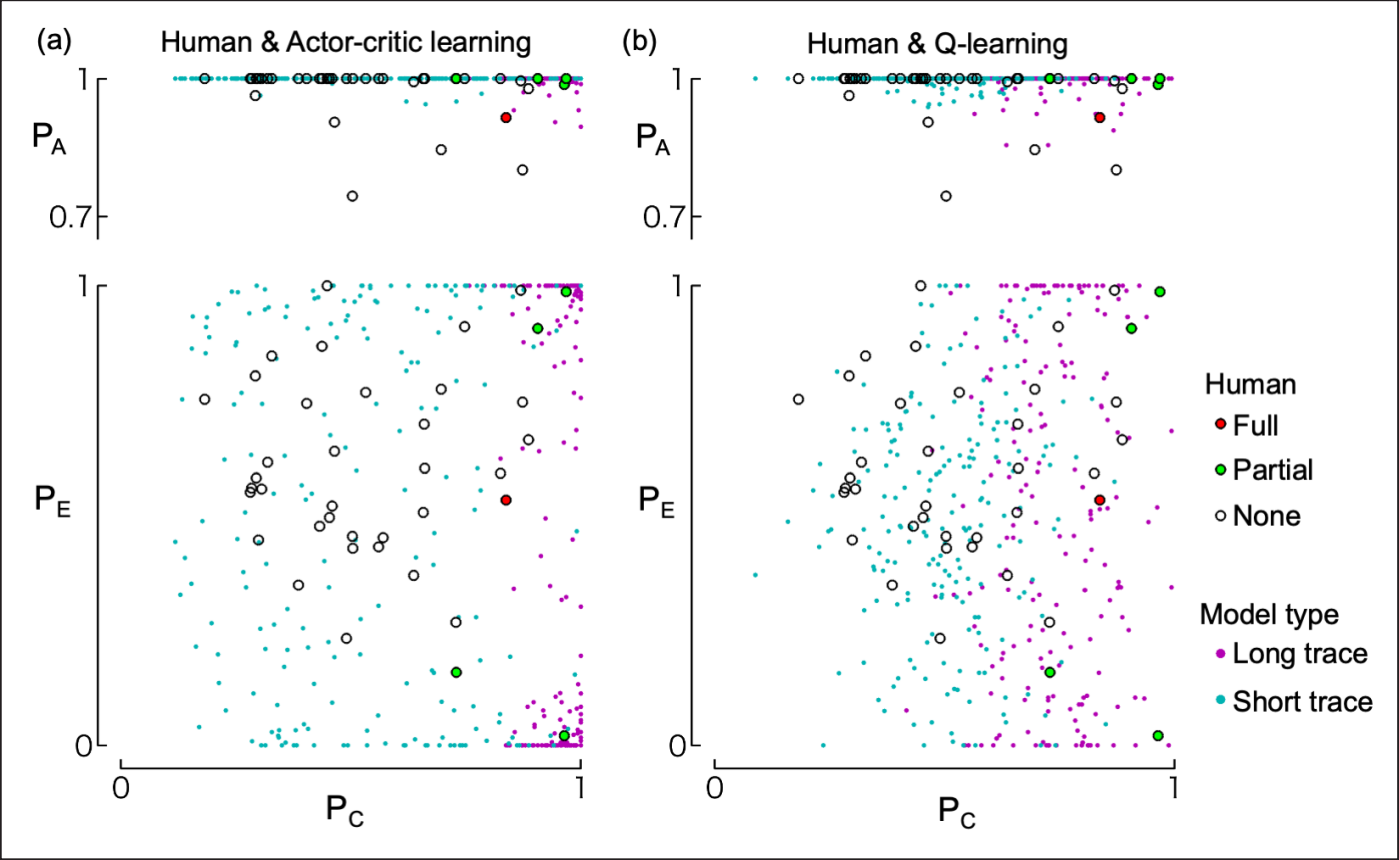
**Distribution of choice rates during the last 30 trials**. The top row shows the relationship between choice rates of C (P_C_) and A (P_A_); the bottom row shows those of C (P_C_) and E (P_E_). Choice rates are weighted averages over the last 30 trials (see *Methods*). Circles represent data from human participants, with different colors indicating their level of understanding of the task structure according to their written reports (i.e., full understanding, partial understanding, or no understanding; see “Written report” section in the *Results*). These human data are identical in panels (a) and (b). Dots indicate the results of 200 simulation runs of the actor-critic model (panel a) and Q-learning model (panel b), using either short-trace (cyan) or long-trace (magenta) learning parameters.

Figure 6 shows the performance of two algorithms (actor-critic and Q-learning with eligibility traces) across four parameter sets (slow learning, high exploration, short trace, and long trace) based on 200 simulation runs, along with histograms of stimulus choices during the final trial bin. The slow learning (Figure 6a, e) and high exploration types (Figure 6b, f) did not show an increase in selection of any stimulus pairs ({A,B}, {C,D}, {E,F}). As all human participants successfully learned to select A, these two types of models differ from the response patterns observed in human participants. The short trace type (Figure 6c, g) showed increased selection of A (associated with an immediate outcome) but failed to increase choices of C (associated with a delayed outcome). Although this type could in theory learn from delayed outcomes due to a non-zero reward prediction value (discount factor in our model), it failed to do so here because the state transitions in our task were independent of the delayed outcomes. In contrast, the long trace type (Figure 6d, h) successfully increased selection of both A and C. In this case, the eligibility traces enabled the association between delayed outcomes and past, outcome-independent state transitions. Figure 7 further illustrates the final choice rates of A, C, and E during the last 30 trials for the short and long trace versions of both actor-critic and Q-learning models. The simulation results are overlaid with human participants’ data for the same indices. As shown, the simulated data largely overlapped with the human data.

### Follow-up test

All of 10 participants met the criterion of two consecutive blocks in which C was chosen at least 8 out of 10 times before the pre-set 501 trials (range: 60–390 trials, median = 105 trials). These results suggest that it is possible to learn the task effectively when provided with information about its underlying structure.

## Discussion

Contrary to our prediction, most participants in the main experiment did not learn to choose the visual stimulus that led to a delayed reward in a later trial. Only nine of 40 participants reached the learning criterion (i.e., eight or more choices of C in two successive 10-trial bins of stimuli C vs D), of whom only one showed a correct understanding of the task in the written report, suggesting explicit understanding of the task. Of the remaining eight participants who reached criterion, four showed partial understanding, whereas the remaining four made no mention of the link between the C vs D trial and E vs F trial in their report. This result suggests implicit understanding, and that what these participants wrote was probably based on retrospective thinking after the test. Caution is called for in interpretation, because eight out of 40 participants showed the opposite-direction bias, i.e., they preferentially chose D, which led to no reward, hence a bias for C or D might simply be due to chance (see also *Supplementary information*).

More than half the participants formed a bias for either E or F despite the absence of any causal link between choice of stimulus and reward. As humans tend to associate an action and an outcome that is temporally close to the action, a bias for E or F in our task might indicate a superstitious belief caused by apparent immediate reward after the trial (Shahar et al. 2019, 2021; Ben-Artzi et al. 2022). It is also noteworthy that seven of 26 participants expressing bias in E vs F trials actually gained more rewards after choosing the less preferred stimulus than the preferred stimulus (see *Supplementary information*). This suggests that the bias was not linked to the rewards overall, but instead emerged at a point during the test and persisted thereafter regardless of whether the preference led to more rewards.

Previous studies using a so-called two-step task have shown that participants generally manifest some level of understanding, either explicit or implicit, about the link between a choice and a delayed outcome (Akam et al. 2015; Daw et al. 2011; Miranda et al. 2020; Walsh et al. 2011). However, in the present study relatively few participants showed understanding of delayed outcome, as judged from their written report after the test. It is unlikely that their poor performance was merely due to irrelevant aspects of the task, such as the images used as visual stimuli, given that most participants quickly learned to select stimulus A over stimulus B (overall, 38 of 40 participants chose A in more than 80% of these trials, and 34 of 40 did so in more than 90%: see also *Supplementary data*). Instead, two aspects of the task structure may underlie this difference. First, in the two-step task, the lag between choice and outcome was consistently one step, whereas in our task, the lag varied from 1 to 5 trials, with a mean of 3 trials. A longer and variable delay likely made it more difficult for participants to detect the action–outcome relationship, rendering our task more challenging than the two-step task. Second, in the two-step task, the sequence of steps was fixed, and the choice at the first step determined the structure of the second step’s outcome. This consistent structure may have facilitated participants’ understanding of the sequence and promoted sensitivity to the delayed outcome. In contrast, our task used a quasi-random trial sequence, where the type of the next trial was independent of the participant’s choice. Moreover, as shown in Figure 2, the trial sequence sometimes contained a series of trials in which the reward in E vs F was determined by the participant’s choice in the second-most recent trial of C vs D, not the most recent (See *Supplementary information* for further analysis of the effect of trial sequence). Importantly, we intentionally designed the task to make explicit detection of the true structure difficult, aiming to encourage a solution based on implicit understanding—specifically, bridging the memory trace of past actions to delayed outcomes. To gain further insights into the ability to link actions with delayed outcomes, the development of additional tasks with varied structures will be necessary.

It is also unlikely that the task exceeded participants’ short-term memory capacities. In Tanaka et al. (2009), participants selected between two visual stimuli, some of which resulted in rewards or punishments delivered three trials later. Their task involved eight stimuli associated with either ±10 or 40 points (Japanese Yen), delivered immediately or after a 3-trial delay. Although it remains unclear exactly how participants understood the contingencies, they demonstrated optimal choice behavior (e.g., preferring the –10-point stimulus over the –40-point stimulus) by the second session (each session included 110 trials). We speculate that the difficulty in our task stems from a combination of humans’ general tendency to prioritize temporally adjacent action–outcome relationships and a propensity to explicitly search for simple rules (e.g., Smith et al., 2018). Additionally, Tanaka et al. (2009) administered the task across multiple sessions and days (110 trials per session × six sessions per day × three days), allowing learning to develop over time. In contrast, we limited our task to 501 trials due to participants’ time constraints. It is possible that with more trials and/or exposure across multiple days, participants would have eventually learned to select C for delayed rewards. Furthermore, offering tangible incentives (e.g., monetary rewards or vouchers) may influence performance.

Our task, as well as those developed by Tanaka et al. (2009) and Sato et al. (2023), is difficult to solve using model-based reinforcement learning because state transitions are independent of actions. Thus, it is likely that model-free learning processes underlie participant behavior, particularly among those who showed an increased rate of C choices, with eligibility traces supporting this learning. However, developing a computational model that captures the full diversity of observed behavior patterns is beyond the scope of the present study. This is especially true as a simple, standard reinforcement learning model cannot explain the behavior of participants who developed a preference for D, which was never reinforced. Nevertheless, constructing a model to account for the diverse behavior patterns observed here is a promising direction for future research. Our primary aim was to develop a simple task that sheds light on state-transition-free learning for delayed outcomes, building on prior work (Sato et al., 2023). Comparing the performance of humans and nonhuman animals on such tasks may further illuminate commonalities and differences in learning processes across species.

Although the task proved difficult for most participants, our simulations demonstrated that learning was possible with certain model-free reinforcement learning algorithms incorporating eligibility traces, given an appropriate set of parameters. It is important to note that the purpose of these simulations was not to argue that participants necessarily used such algorithms, nor to determine the best-fitting parameters, but rather to verify whether a reinforcement-learning-based agent could learn to select the stimulus associated with the delayed reward in our task. The parameters we used were selected arbitrarily. Nonetheless, the overlap between the simulation data and human behavior (as shown in Figure 7) suggests that our assumption about the feasibility of reinforcement learning in this context is reasonable. Moreover, our follow-up experiment showed that participants could learn the task when provided with prior information about its structure. Together, these findings indicate that the task is not inherently unsolvable. In the absence of explicit instruction, one possible reason for the difficulty is that participants may have constructed incorrect mental models of the task. Prior studies on two-step tasks (e.g., Daw et al., 2011) have shown that humans often exhibit a mixture of model-free and model-based behaviors. Although our simulation used a purely model-free framework, it is likely that participants’ behavior reflected a blend of strategies. Post-experiment reports (see *Supplementary data*) revealed that some participants mistakenly assumed that features such as stimulus location or shape predicted rewards, reflecting incorrect model-based assumptions that may have interfered with learning.

Working memory may also have played a role in task performance. Participants rapidly learned to select stimuli associated with immediate rewards, suggesting that they could discriminate between stimuli and maintain these associations in working memory. However, the obscured temporal relationship between stimuli and delayed rewards likely increased memory demands, possibly contributing to the emergence of selection patterns unrelated to the actual task structure. This interpretation aligns with findings from Ben-Artzi et al. (2022), who showed that high working memory load can promote outcome-irrelevant learning, in which participants repeat behaviors unrelated to the actual outcome contingencies.

In conclusion, at least some humans have the ability to link an action to its outcome even if there is a lag between these, as shown in the current cognitive task involving delayed feedback and a state-transition-free structure. Also, our dataset revealed marked individual differences in people’s explicit and implicit understanding of the link between an action and delayed outcome, formation of bias with no causal relationship with a reward but in close proximity to reward, and persistence of choice that actually led to fewer rewards. Two potential future directions, among others, can be considered. The first is to investigate factors underlying these individual differences. The second is to apply sophisticated models incorporating various factors such as reward sensitivity and behavioral persistency to explain the full range of human behavior in this task.

## Data Accessibility Statement

Supplementary information, Supplementary data and related files are available on the Open Science Framework (OSF): https://osf.io/prt84/.

Additional data, materials and codes are available from the corresponding author, S.H., upon reasonable request.
